# Rectovesical Fistula Related to Transurethral Resection of a Bladder Lesion

**DOI:** 10.1155/2011/801290

**Published:** 2011-09-29

**Authors:** Ramazan Topaktaş, Abdulkadir Tepeler, Omer Kurt, Mehmet Remzi Erdem, Abdullah Armağan, Şinasi Yavuz Önol

**Affiliations:** ^1^Department of Urology, Faculty of Medicine, BezmialemVakif University, Istanbul, Turkey; ^2^Department of Urology, Bayrampasa Community Hospital, Istanbul, Turkey

## Abstract

A rectovesical fistula (RVF) is an uncommon complication of urooncologic surgery. Although several RVFs have been reported, our case is the first reported RVF in the literature that iatrogenically occurred after transurethral resection of the bladder. A single-stage primary repair with omental flap interposition without a colostomy was successfully performed because of the persistence of the fistula during followup. After 6 months of followup, no fistula or bladder mass was detected.

## 1. Introduction

A rectovesical fistula (RVF) is usually seen secondary to diverticulae, inflammatory bowel diseases [1], trauma, perirectal abscesses, iatrogenic injury, malignancy, radiotherapy, or chemotherapy [2, 3].

Although rectovesical fistulae have been reported after extirpative or ablative procedures of the prostate for both benign and malign prostatic diseases [4], no case of RVF after transurethral resection of the bladder (TURB) had been previously reported. Mucosal injuries or a perforation of the bladder are also common complications of endourologic surgeries. To the best of our knowledge, this is the first case of RVF after TURB in the literature, which was managed with separate closure of three layers without a colostomy. 

## 2. Case Presentation

**Figure 1:**
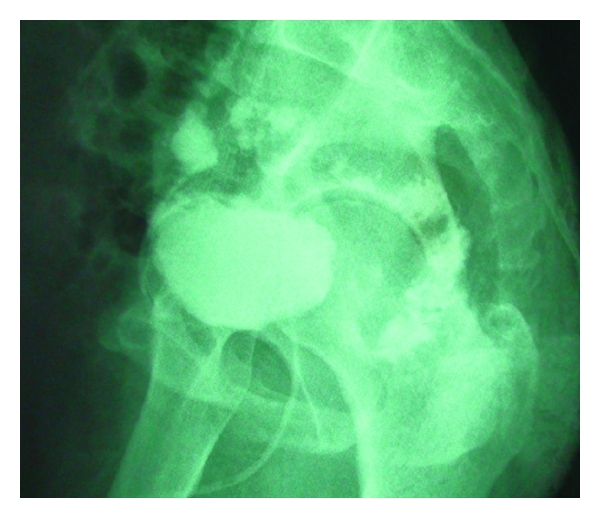
Preoperative oblique cystography shows extravasation of contrast material through the fistula from bladder to the rectum.

**Figure 2:**
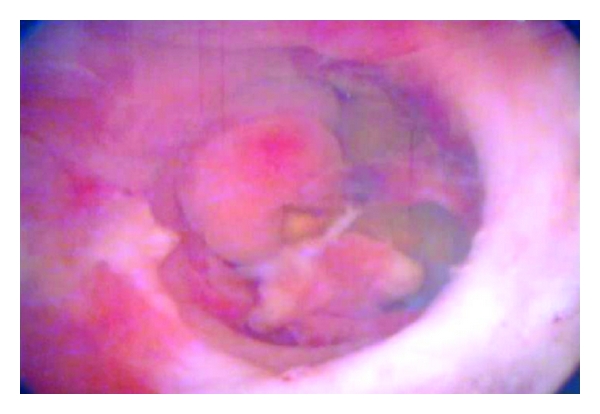
Preoperative cystoscopic evaluation of the resection area showing the rectal lumen following an iatrogenic rectal injury.

A 56-year-old man who underwent a transurethral resection for a 2 × 2-cm papillary bladder lesion defined on ultrasound was referred to our clinic with a suspicion of an RVF at the first day after surgery due to newly defined complaints of urine leakage from the rectum, fecaluria, pneumaturia, and suprapubic pain. A physical examination revealed suprapubic tenderness without peritoneal findings. Pathology of the specimen revealed cystitis cystica. No other significant medical history was evident. A urethral catheter was placed, intravenous broad spectrum antibiotics were immediately initiated, and then he was hospitalized. Extravasation of contrast medium through the fistula to the rectum was observed on oblique cystography (Figure 1). Cystoscopically a 1 cm fistula orifice was disclosed in the left posterior wall of the bladder (Figure 2), but both ureteral orifices were intact. The first management approach included placement of an indwelling urethral catheter, administration of antibiotics (cefoperazone-sulbactam and metronidazole), cessation of enteral nutrition, and commencement of parenteral nutrition. After a 15-day follow-up period, the pneumaturia persisted, and a second-look cystoscopy showed the fistula orifice; consequently, immediate surgery was performed. 

**Figure 3:**
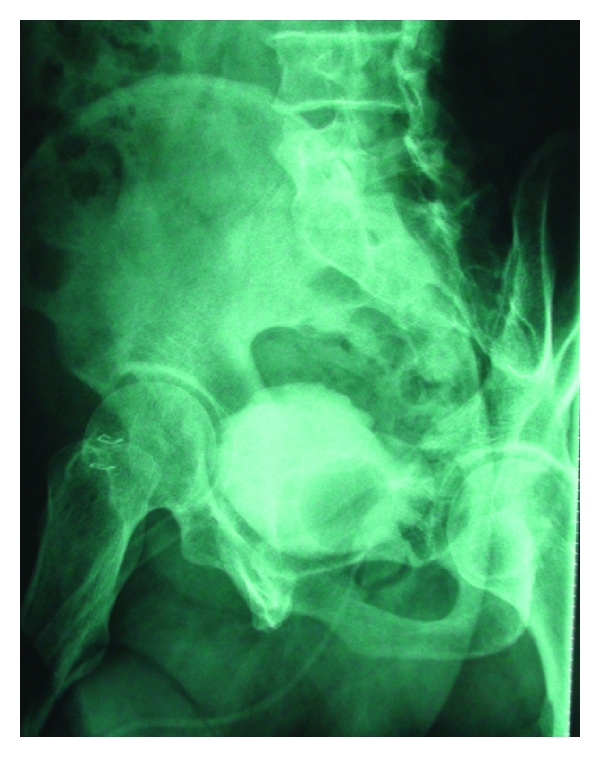
Postoperative oblique cystography with no extravasation.

**Figure 4:**
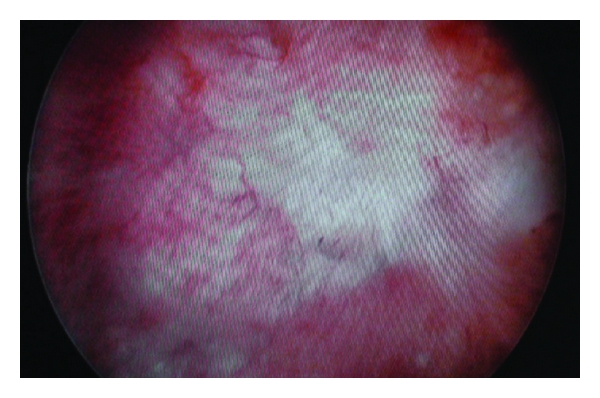
Postoperative cystoscopy with no fistula.

During surgery with a midline infraumbilical incision, the posterior bladder wall, lower segment of the left ureter, anterior rectal wall, and the fistula tract were clearly exposed. The fistula tract and its surrounding tissue seemed nonviable. This necrotic tissue and the fistula tract on both sides of the bladder and rectum were excised with 3-4 mm of healthy tissue. The anterior rectal wall, perirectal area, and surrounding tissues were closed separately in three layers. The posterior bladder wall was closed watertight, and a 20 Fr urethral Foley was inserted. An omental pedicle flap with vascular supply was placed between the rectum and bladder to enhance restoration. Broad-spectrum antibiotics were continued for 5 days after surgery. A liquid diet was started on postoperative day 1, and a regular low-fiber diet was started on day 3. Anal dilation was performed for fecal discharge. The patient recovered uneventfully and was discharged on postoperative day 4. The urethral catheter was removed on postoperative day 14 after normal cystographic findings (Figure 3). A urinalysis and cultures were negative during the first, third, and sixth months. Postoperative cystoscopy (Figure 4) was performed at the first month and then the patient was followed up for 6 months with the help of symptoms and urine tests. Normal urine tests and cessation of symptoms disclosed improvement of the fistula. 

## 3. Discussion

An enterovesical fistula is a rare disease, with an estimated two to three patients per 10 000 hospital admissions and an annual incidence of 0.5 per 100 000 [2]. Clinical findings of RVF include urine leakage from the rectum, pneumaturia, and fecaluria [4–6]. The definitive diagnosis is clarified by visualizing the fistula tract and orifices by cystography and an endoscopic evaluation of the bladder and rectum. 

Several approaches from conservative management to complex procedures could be used based on the fistula tract. Initial management should include placement of an indwelling urethral catheter and administration of broad-spectrum antibiotics. Thurairaja presented a 46-year-old man suffering from RVF after falling onto a chair. He was conservatively managed with antibiotics for 3 weeks and with urinary diversion using a suprapubic catheter for 5 weeks. At the eighth week of followup, a computed tomography scan without administration of contrast material showed no gas in the bladder [7]. The 2-week conservative management was unsuccessful in our patient, so we decided to perform surgery. 

Several surgical procedures have been described, and controversy exists about the best treatment method for RVF repair [6]. These include simple surgical repair, a laparotomy, or fecal or urinary diversion [8]. Walker and Bowen reported a transvesical approach to traumatic RVF. The bladder was opened longitudinally from the anterior wall; the posterior defect was visualized clearly, then perirectal tissues were repaired in two layers. A colostomy was also performed [9]. Recently, Chiang et al. reported a nonsurgical minimally invasive treatment option for traumatic RVF. They controlled the fistula by applying hemoclips to the fistula limbs rectoscopically [1]. No fistula was reported in the followup of the patient. 

RVF can be repaired by open, laparoscopic, or robotic methods. Renschler and Middleton described the open York-Mason technique for RVF repair [10]. Open repair is superior to endoscopic techniques in terms of operation time. Sotelo et al. also reported both laparoscopic [8] and robot-assisted [6] approaches. Endoscopic methods have the advantage of a short hospital stay. Success rates for all these techniques are similar, but endoscopic studies include fewer patients [6, 8]. 

 Smith and Veenema [11] and McLaughlin and McCullough [12] suggested the necessity of preoperative bowel preparation for rectal closure and did not consider a colostomy. In contrast, Borland reported that an omental flap provided safe primary rectal repair without a colostomy in the absence of preliminary complete bowel preparation [13]. Closure of a fistula with multiple layers provides a high success rate without a colostomy, as in Borland's outcome. As mentioned in the literature, meticulous three-layer rectal repair of the anterior rectal wall, perirectal tissues, and local tissues allows a safe closure of the fistula without a diversion colostomy as in our case. Our technique was safer, less invasive, and more effective by avoiding the morbidity of a diverting colostomy for managing iatrogenic RVF. Furthermore, similar results have been reported in the literature.

 In conclusion, RVF is a dreaded complication of urooncological surgery. Single-stage primary repair with omental flap interposition is a sufficient and safe method to treat RVF with no additional morbidity originating from a diverting colostomy.
